# Use of Bacterially Expressed dsRNA to Downregulate *Entamoeba histolytica* Gene Expression

**DOI:** 10.1371/journal.pone.0008424

**Published:** 2009-12-23

**Authors:** Carlos F. Solis, Julien Santi-Rocca, Doranda Perdomo, Christian Weber, Nancy Guillén

**Affiliations:** 1 Institut Pasteur, Unité Biologie Cellulaire du Parasitisme, Paris, France; 2 INSERM U786, Paris, France; INSERM U567, Institut Cochin, France

## Abstract

**Background:**

Modern RNA interference (RNAi) methodologies using small interfering RNA (siRNA) oligonucleotide duplexes or episomally synthesized hairpin RNA are valuable tools for the analysis of gene function in the protozoan parasite *Entamoeba histolytica*. However, these approaches still require time-consuming procedures including transfection and drug selection, or costly synthetic molecules.

**Principal Findings:**

Here we report an efficient and handy alternative for *E. histolytica* gene down-regulation mediated by bacterial double-stranded RNA (dsRNA) targeting parasite genes. The *Escherichia coli* strain HT115 which is unable to degrade dsRNA, was genetically engineered to produce high quantities of long dsRNA segments targeting the genes that encode *E. histolytica* β-tubulin and virulence factor KERP1. Trophozoites cultured in vitro were directly fed with dsRNA-expressing bacteria or soaked with purified dsRNA. Both dsRNA delivery methods resulted in significant reduction of protein expression. In vitro host cell-parasite assays showed that efficient downregulation of *kerp1* gene expression mediated by bacterial dsRNA resulted in significant reduction of parasite adhesion and lytic capabilities, thus supporting a major role for KERP1 in the pathogenic process. Furthermore, treatment of trophozoites cultured in microtiter plates, with a repertoire of eighty-five distinct bacterial dsRNA segments targeting *E. histolytica* genes with unknown function, led to the identification of three genes potentially involved in the growth of the parasite.

**Conclusions:**

Our results showed that the use of bacterial dsRNA is a powerful method for the study of gene function in *E. histolytica*. This dsRNA delivery method is also technically suitable for the study of a large number of genes, thus opening interesting perspectives for the identification of novel drug and vaccine targets.

## Introduction

*Entamoeba histolytica* is a protozoan parasite causing human amoebiasis, a disease that is a public health problem in endemic areas [1], [2]. Amoebiasis is transmitted by the ingestion of cysts present in food or water contaminated with feces from amoebic individuals. The disease is usually asymptomatic; however some patients develop the symptomatic invasive form leading to dysentery and liver abscesses that can result in a fatal outcome without medication [1], [2]. The treatment of the disease relies only in a reduced array of drugs [3]. Recent studies have shown that *E. histolytica* cultured *in vitro* can develop resistance against these drugs, urging the need for new therapeutic or prophylactic tools to control this parasitic disease [4]. These developments will not be possible without a deeper understanding of *E. histolytica* physiology and pathogenic process.

Experimental studies on animal models and *ex vivo* procedures have so far provided vital insights into the amoebic pathogenic process [5]–[11]. However, until now only a few amoebic factors have been characterized as playing a major role during the pathogenic process. These include: (i) the Gal/GalNAc-inhibitable lectin [10], [11], (ii) the lysine-rich protein KERP1 [12], (iii) the cysteine proteinase CP5 [13], (iv) the GPI-anchored surface components [14] and (v) the lipopeptidophosphoglycans (EhLPPG) [15], [16]. Therefore, further progress is necessary in the field of amoebiasis towards the establishment of procedures combining the use of experimental models and molecular tools capable of knocking down gene expression that would allow more efficient and systematic studies.

The Entamoeba consortium recently reported the whole genome of *E. histolytica*, predicting 8,343 genes [17]. However, a major drawback for molecular genetics in *E. histolytica* is that this parasite is not amenable to standard gene-replacement procedures due to an undetermined genome ploidy and sexual cycle. RNA interference (RNAi) has recently emerged as a powerful tool to knockdown gene expression, especially in eukaryotic organisms that are not amenable to standard gene-replacement procedures [18], [19]. RNAi is a highly conserved eukaryotic pathway for gene silencing that is present from plants to humans. The pathway is triggered by long double-stranded RNA (dsRNA) that is cleaved by an endoribonuclease called Dicer into small interfering RNA (siRNA) oligonucleotides. The resulting siRNA assemble within endoribonuclease-containing complexes known as RNA-induced silencing complexes (RISCs) that will cleave and destroy the cognate RNA.

Although little is known about RNAi machinery in *E. hystolytica*, some orthologous proteins have recently been characterized as sharing similarities with other eukaryotic RNAi factors [20]. Moreover, our group previously reported efficient gene silencing for *E. histolytica* trophozoites (vegetative stage of the parasite) soaked with chemically synthesized siRNA oligonucleotides [21]. Other pioneer reports also showed efficient gene silencing in trophozoites transformed with plasmid constructs designed for episomal expression of antisense and short hairpin RNA (shRNA) [22]–[24].

Here we explored the feasibility of a direct and cost-effective approach for the delivery of long dsRNA segments. This method takes advantage of intrinsic phagocytic properties of *E. histolytica*, which naturally feeds on probiotic bacteria present in the intestinal flora. We showed that trophozoites either directly fed with dsRNA-expressing bacteria, or soaked with purified dsRNA resulted in down-regulation of gene expression. *In vitro* functional assays showed that downregulation of the virulence factor KERP1 mediated by bacterial dsRNA reduces parasite adhesion to human cells. Moreover, initial studies using a large repertoire of bacterial dsRNA targeting amoeba genes led to the identification of three genes potentially playing a role in the growth of the parasite. Our observations suggest that genetic interference mediated by bacterial dsRNA is a convenient approach to conduct large-scale studies for the analysis of gene function in *E. histolytica*.

## Results

### Double-Stranded RNA Bacterial Feeding Targeting *E. histolytica* β-Tubulin

Based on *E. histolytica* phagocytic properties, initial experiments were conducted to verify whether delivery of bacterial dsRNA targeting amoeba genes would produce changes in transcript and protein levels. Initial gene silencing studies were carried out using *E. histolytica β*-tubulin (GenBank accession number EHI_049920 or EHI_167010) as a target gene since a highly specific antibody and quantitative PCR tools were already available in our laboratory to characterize the outcome of the phenotype (see Material and Methods).

**Figure 1:**
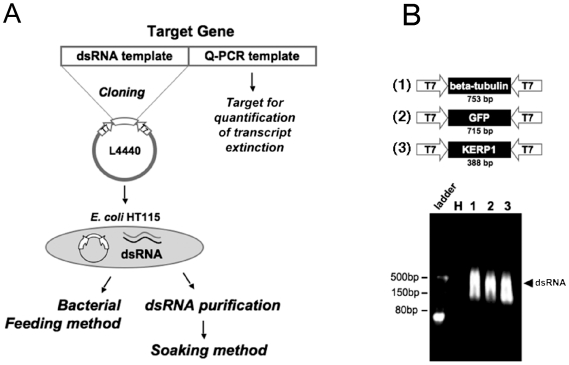
Genetic interference following ingestion of dsRNA-expressing bacteria by *Entamoeba histolytica*. A. General scheme for dsRNA production. The 5′-terminal portions of the genes were amplified by PCR and cloned into the bacterial vector L4440 for dsRNA transcription. Primers used for quantification of mRNA by qRT-PCR match with the 3′-terminal part of the gene, which was not cloned into the plasmid (marked “Q-PCR template” in the figure), allowing the exclusive quantification of genome-encoded, sense-orientated transcripts. The multicloning site of L4440 vector is bidirectionally flanked by T7 promoters driving the synthesis of RNA complementary strands (*i.e*. dsRNA). The recombinant plasmid was transfected into the bacterium HT115, allowing dsRNA purification in order to conduct the parasite soaking experiments or direct feeding of parasites with the expressing bacteria. B. Constructs performed in this work. Three independent DNA fragments were cloned and used for RNAi experiments including β-tubulin-, KERP1- and GFP-encoding genes (upper panel). Upon production of dsRNA, a nuclease-resistant dsRNA was detected in lysates of the recombinant bacterium (bottom panel).

*Escherichia coli* strain HT115 was transformed with the plasmid constructs L4440-beta-tubulin and L4440-GFP that were designed for the expression of long dsRNA segments (Fig. 1). Bacterial dsRNA expression was analyzed upon induction as previously described [25]. Total bacterial nucleic acids were purified and treated with DNase and RNaseA to degrade DNA and single-stranded RNA. Nucleic acid samples were analyzed by agarose gel electrophoresis (Fig. 1B). A massive smear of dsRNA was observed with predominant dsRNA bands around the expected sizes for all transformants except for the untransformed wild-type HT115 bacteria. The variation between the theoretical and observed size of purified products may be due to the presence of secondary structures in the RNAs, exposing single-stranded fragments within the target during digestion, and also affecting migration in gel, as already commented [25].

**Figure 2:**
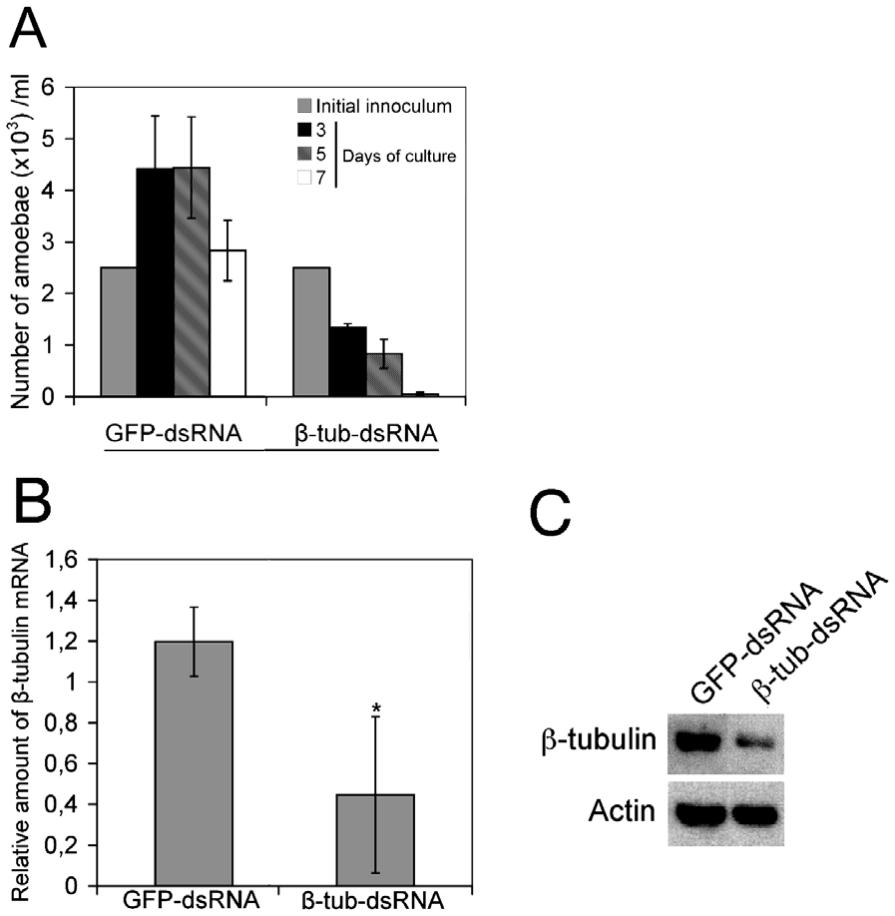
Targeting β-tubulin-encoding gene by dsRNA expressed in bacteria. Following 3, 5 and 7 days of culture of 3×10^5^ *E. histolytica* trophozoites in the presence of bacteria expressing dsRNA from genes encoding GFP (control) or β-tubulin (test), the parasites were counted under the microscope(A). At day 3 post-inoculation, endogenous mRNA was quantified by qRT-PCR (B) and protein levels were observed by western blot (C) according to procedures described in the materials and methods. Each graph represents the mean of 3 independent experiments.

Trophozoites cultured under standard *in vitro* conditions were fed with bacteria (10^3^, 10^4^, and 10^5^ bacteria per amoeba) producing dsRNA. By this setting up, we determined that 10^4^ bacteria per amoeba was the optimal dose to observe effects on growth with β-tubulin targeting. After inoculation of bacteria, growth was followed for 3, 5 or 7 days (Fig 2A), revealing that growth was affected in β-tubulin samples from the third day of culture. In the control condition, i.e. with bacteria expressing *gfp* dsRNA, the maximum number of trophozoites was detected as soon as the third day post-inoculation. Thus, we retained this condition (inoculum and time) for the following experiments.

Targeting of β-tubulin resulted in a significant reduction of fifty-eight percent of the endogenous transcript as determined by real-time PCR. Gene silencing appeared to be highly specific since no reduction of the β-tubulin transcript was detected in trophozoites fed with control bacteria carrying dsRNA from the GFP-encoding gene (Fig. 2B). Reduction of β-tubulin transcript also led to a qualitative decrease of protein levels, as detected by western blot analysis using a specific antibody (Fig. 2C).

### Targeting of KERP1 Virulence Factor by dsRNA

**Figure 3:**
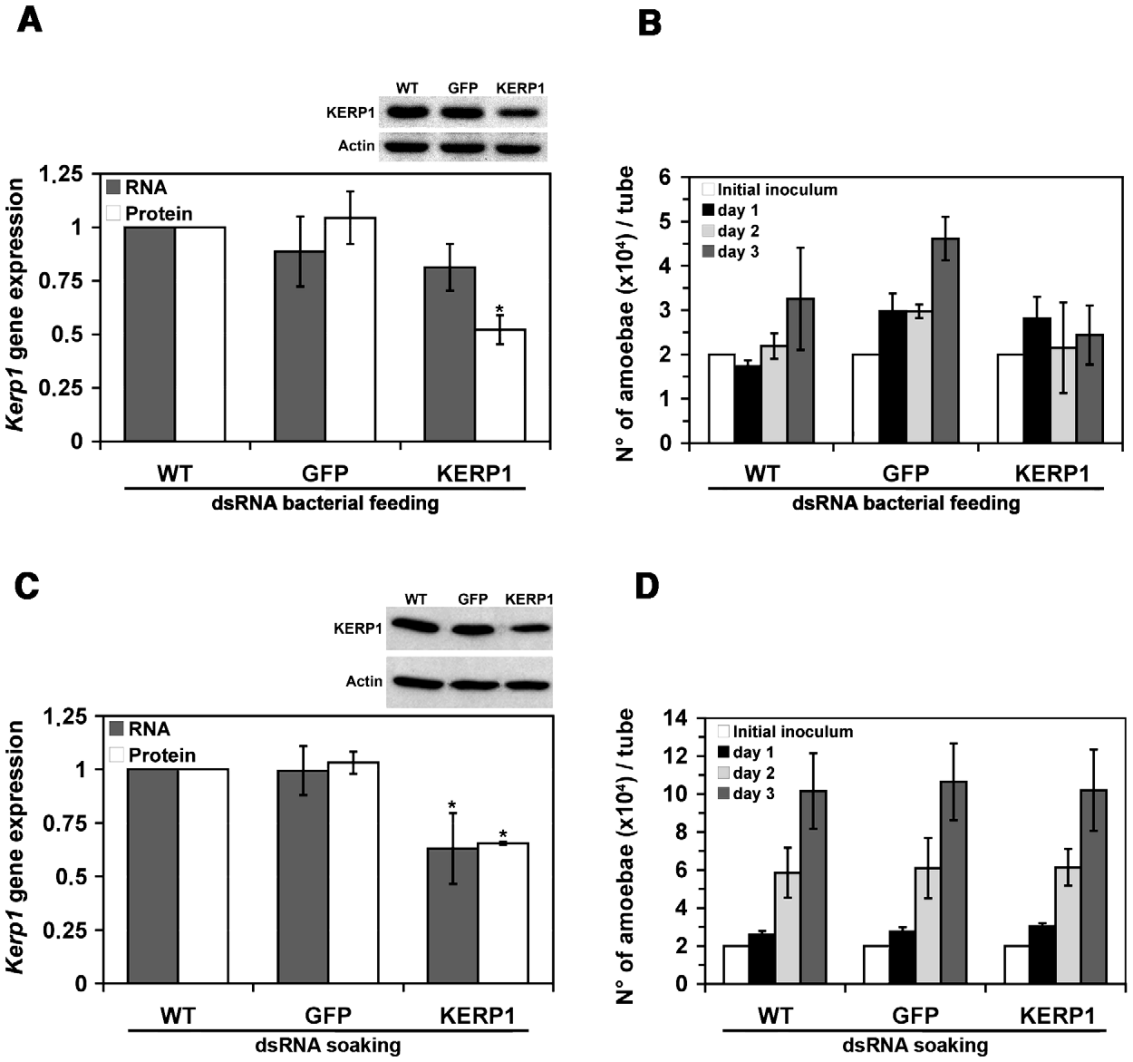
Phenotype of trophozoites fed with bacteria producing *kerp1* dsRNA or soaked in medium containing *kerp1* dsRNA. Experiments were performed with trophozoites incubated without (wild type, “WT”), with bacteria producing *gfp* or *kerp1* dsRNAs or with purified dsRNA (50 µg/ml) for the indicated times. A and C. Upper frames: Western blot analysis of KERP1 protein abundance after 2-day incubation. Actin immunodetection confirms that equal numbers of amoebae were loaded in each well. Lower frames: Quantification of *kerp1* gene mRNA expression and protein abundance within trophozoites after feeding for 2 days. Quantitative PCRs were normalized by quantifying the mRNA coding for L9 ribosomal protein. Western blots were normalized using immunodetection of actin. For both methods, relative quantification of the samples was achieved by linear regression using dilutions of samples as standards. Data were calculated from 3 independent experiments. B and D. Analysis of parasite growth. Tubes were inoculated with 2×10^4^ trophozoites and 2×10^7^ bacteria, when applicable. After 1 to 3 days of interaction, amoebae were harvested and counted with a hemocytometer. Data were calculated from 3 independent experiments (*n* = 3).

After observing specific gene silencing of an essential gene such as β-tubulin, we were interested in determining whether the phenomenon of RNAi could be inferred for other genes. *E. coli* strain HT115 was thus transformed with plasmid construct L4440-KERP1 that was designed for the expression of dsRNA targeting *E. histolytica* virulence factor KERP1 [12] and dsRNA expression was verified as described above (Fig. 1). Trophozoites cultured *in vitro* were fed with dsRNA-expressing bacteria targeting KERP1 using a ratio of 10^4^ bacteria per amoeba. After two days of bacterial feeding, trophozoite crude extracts and mRNA samples were prepared to characterize the gene expression profile. A representative western blot is shown in Figure 3A; together with two other independent experiments, it allowed an evaluation of protein abundance by semi-quantitative densitometry analysis of scanned films. An average decrease of forty-eight percent of protein was determined, although no significant reduction of endogenous transcript was observed. Trophozoites fed with *gfp* dsRNA-expressing bacteria showed higher growth rates at day one compared to the wild-type control (p = 0.032; Fig. 3B). Nevertheless, in all the other samples, we could not detect any significant variation in trophozoite number between the three tested conditions. To reduce the possible sources of variation impacting the reproducibility of the experiment, trophozoites were directly soaked with purified dsRNA.

The gene expression profile was also characterized as above for trophozoites soaked with different concentrations of purified bacterial dsRNA targeting KERP1. A thirty-four percent reduction of protein was observed by using 50 µg of purified *kerp1* dsRNA per mL of culture media (Fig. 3C), whereas incubation with 5 µg/ml and 25 µg/ml *kerp1* dsRNA did not trigger a sensible reduction of KERP1 abundance (data not shown). Moreover, dsRNA soaking resulted in a consistent reduction of *kerp1* transcript as opposed to the feeding method (Fig. 3C). Growth rate identical to untreated cells (Fig. 3D) was also observed.

**Figure 4:**
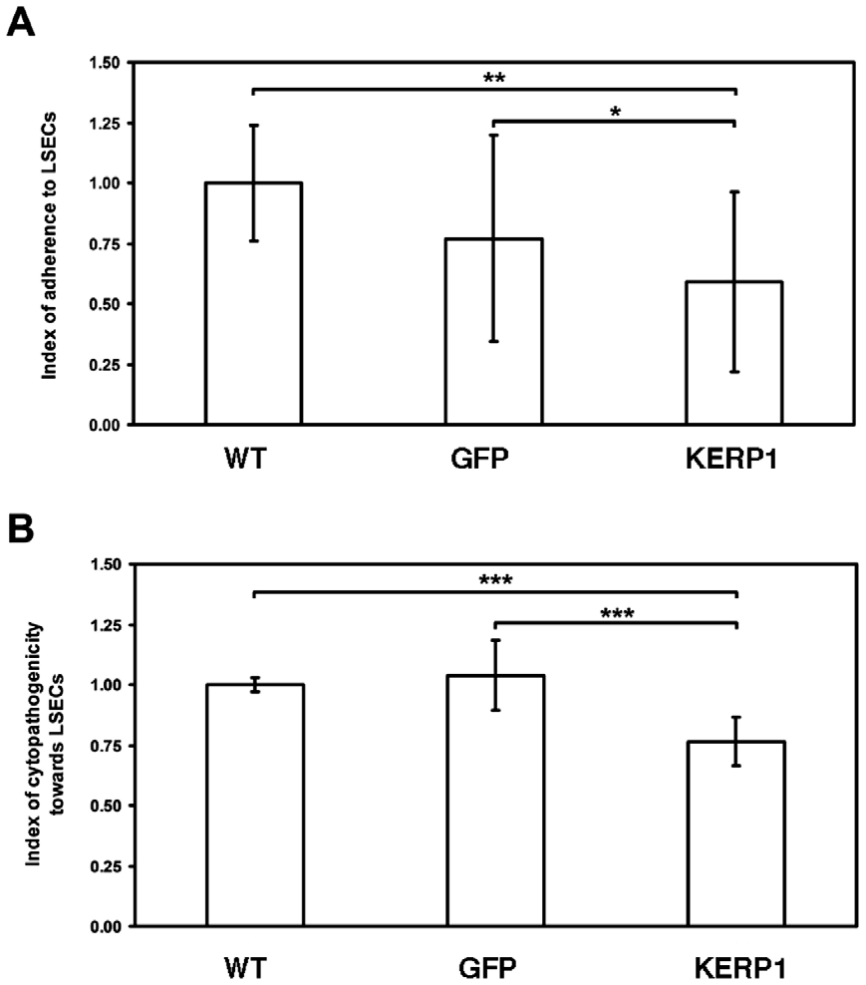
Study of KERP1 involvement in trophozoite interaction with LSECs. A. Assay for adherence of modified trophozoites to LSECs. Quantification of trophozoites bound to LSECs after 30 min contact showed 50% decrease in adherence of KERP1-reduced trophozoites. Data were calculated from 3 independent experiments (*n* = 3). B. Assay for cytopathogenicity of modified trophozoites on LSECs. Quantification of fluorescence emitted by LSECs after 1 hr incubation with trophozoites showed a decreased cytopathogenicity of KERP1-reduced trophozoites leading to 24% reduction in fluorescence. Data obtained from 3 independent experiments (*n* = 3).

*In vitro* parasite–human cell interaction assays were carried out to support the effectiveness of gene silencing mediated by bacterial dsRNA. According to the possible interference of bacteria in adherence assays, we chose to use exclusively the soaking method to perform the following experiments. Trophozoites that were previously soaked with *kerp1* dsRNA during forty-eight hours, adhered significantly less (0.59±0.37 relative units [r.u]) to fixed human liver sinusoidal cells (LSECs) compared to untreated trophozoites (WT; 1.00±0.24 r.u., p = 1.46×10^−3^) or trophozoites soaked with *gfp* dsRNA (GFP: 0.77±0.43 r.u., p = 2.47×10^−2^), as presented in Figure 4A. In addition, trophozoites soaked with *kerp1* dsRNA showed a lower cytopathogenicity towards LSEC monolayers compared to controls (*kerp1* dsRNA: 0.76±0.10 r.u.; WT: 1.00±0.03 r.u., p = 2.47×10^−4^; GFP: 1.04±0.15 r.u., p = 4.28×10^−4^; Fig. 4B).

### Identification of Genes Potentially Implicated in the Growth of *E. histolytica*

**Figure 5:**
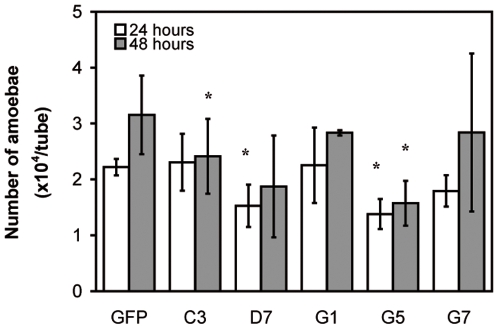
Selection of candidate genes for potential role in growth defects in *E. histolytica* by feeding experiments. Quantification of trophozoite growth was performed at 24 and 48 hrs during the feeding experiment with the C3, D7, G1, G5 and G7, previously selected from a visual screening of trophozoites soaked with 85 target genes. Results are the mean of 3 independent experiments (*n* = 3).

We explored the use of dsRNA for gene expression interference for a random identification of genes whose inactivation can be essential for developmental phenotype of trophozoites cultured under axenic conditions. To this goal, we prepared a cDNA library (in L4440 plasmid vector, using HT115 bacterial strain), sequenced the bacterial clone inserts and compared the data to the *E. histolytica* annotated genome by BLAST. A repertoire of 408 distinct amoebic DNA sequences was obtained, with 256 of them corresponding to unique genes. Eighty-five ORFs encoding potential proteins of unknown function (Table 1) were identified and assessed for dsRNA gene silencing. Trophozoites cultured in 96-well microtiter plates were individually treated with fractions of distinct dsRNA species that were purified from bacterial microtiter plate inductions. After twenty-four and forty-eight hours of treatment with purified dsRNA fractions, light micrographs from microtiter plate wells were obtained for the identification of clones potentially leading to a reduction or inhibition of trophozoite growth compared to untreated or *gfp* dsRNA control conditions. Five dsRNA fractions targeting amoebic genes resulted in microscope visible reduction of trophozoite numbers (data not shown); the corresponding bacterial clones were called C3, D7, G1, G5 and G7. The feeding method was used to further characterize the growth phenotypes of these five clones. Trophozoites cultured in standard culture tubes were individually fed with bacteria expressing the dsRNA species from the clones. A significant delay in trophozoite growth at 24 hours was confirmed with bacterial clones expressing dsRNA targeting annotated genes EHI_176840 (clone D7) and EHI_148060 (clone G5; Fig. 5B and Table 2) and at 48 hours with EHI_053430 (clone C3) and EHI_148060 (clone G5). *In silico* analysis of these three currently uncharacterized target genes, showed that EHI_176840 encoded a protein containing a ribonucleotide-binding domain characteristic of Ribonucleases II (RNP), EHI_148060 encoded a protein containing a transmembrane domain (DUF850) and EHI_053430 has a domain homologous to ubiquitin. Utilizing a t-paired statistical test showed that the clone G7, expressing dsRNA targeting EHI_178560 (carrying a lipid-binding domain), was not significant although a sensible reduction in parasite growth was observed at 24 hours post RNAi treatment. The target gene knockdown shown by these data suggest that dsRNA induces downregulation of amoebic gene expression whether dsRNA was delivered by feeding of bacteria or by soaking parasites in purified dsRNA.

**Table 1:** Genes encoding proteins reffered as hypothetical cloned in the L4440 plasmid.

Library Clone	Gene ID*	Match %	length bp	Mis match	Gap	Start gene	End gene	E Value	Score
ehcs0010A05.b	EHI_194230	100	316	0	0	750	435	3E-142	502
ehcs0010A13.b	EHI_079850	100	363	0	0	1	363	2E-161	565
ehcs0010A23.b	EHI_048560	100	266	0	0	8	273	9E-119	423
ehcs0010B04.b	EHI_159490	100	165	0	0	1	165	1E-70	263
ehcs0010B11.b	EHI_012120	99,15	704	5	1	12	715	0	1090
ehcs0010B18b	EHI_010710	100	175	0	0	792	966	1E-75	278
ehcs0010B23.b	EHI_023620	100	131	0	0	1558	1428	1E-54	209
ehcs0010C19.b	EHI_153280	100	75	0	0	581	655	4E-22	101
ehcs0010C22.b	EHI_156420	98,49	730	8	2	56	785	0	1012
ehcs0010D11.b	EHI_147520	100	296	0	0	84	379	3E-129	458
ehcs0010D15.b	EHI_007620	100	170	0	0	179	10	3E-73	271
ehcs0010D17.b	EHI_189900	100	259	0	0	1272	1530	5E-115	412
ehcs0010E16.b	EHI_044370	100	294	0	0	1891	1598	5E-132	467
ehcs0010E24.b	EHI_153520	100	133	0	0	60	192	6E-29	123
ehcs0010F11.b	EHI_021360	100	480	0	0	9	488	0	762
ehcs0010F23.b	EHI_159750	100	183	0	0	555	737	2E-79	291
ehcs0010G03.b	EHI_138770	100	296	0	0	1	296	5E-112	400
ehcs0010G15.b	EHI_136410	100	661	0	0	1	661	0	1036
ehcs0010H14.b	EHI_060360	99,71	689	2	0	374	1062	0	1015
ehcs0010H15.b	EHI_119930	100	333	0	0	2013	1681	1E-150	529
ehcs0010H21.b	EHI_053430	99,49	588	3	0	39	626	0	871
ehcs0010H23.b	EHI_001130	100	109	0	0	1279	1387	9E-25	109
ehcs0010I11.b	EHI_014250	100	386	0	0	206	591	4E-96	348
ehcs0010I16.b	EHI_024460	99,55	224	1	0	2004	1781	3E-98	355
ehcs0010I17.b	EHI_030450	100	624	0	0	2102	1479	0	811
ehcs0010I22.b	EHI_026400	100	473	0	0	1171	699	0	751
ehcs0010J01.b	EHI_146390	100	104	0	0	6	109	2E-12	68,2
ehcs0010J12.b	EHI_186830	100	157	0	0	186	30	5E-67	250
ehcs0010J14.b	EHI_107270	100	579	0	0	5	583	0	908
ehcs0010J20.b	EHI_166860	100	320	0	0	333	14	2E-144	508
ehcs0010K07.b	EHI_158130	99,7	335	0	1	356	23	5E-110	394
ehcs0010K16.b	EHI_012280	100	673	0	0	676	4	0	801
ehcs0010K17.b	EHI_141940	100	387	0	0	22	408	3E-176	614
ehcs0010K19.b	EHI_146110	98,98	683	7	0	986	304	0	1061
ehcs0010K23.b	EHI_010570	99,86	697	1	0	20	716	0	1103
ehcs0010L01.b	EHI_063570	100	306	0	0	7	312	9E-138	486
ehcs0010L05.b	EHI_045540	100	388	0	0	434	47	9E-177	616
ehcs0010L10.b	EHI_148060	100	334	0	0	461	794	4E-151	530
ehcs0010L20.b	EHI_125630	100	408	0	0	4	411	0	635
ehcs0010L21.b	EHI_049670	100	58	0	0	2401	2458	2E-19	93,5
ehcs0010L24.b	EHI_050800	100	192	0	0	1704	1513	2E-83	305
ehcs0010M11.b	EHI_113930	100	84	0	0	1	84	4E-32	134
ehcs0010N05.b	EHI_198680	100	328	0	0	488	161	2E-131	465
ehcs0010N11.b	EHI_042710	100	162	0	0	515	676	2E-69	258
ehcs0010N13.b	EHI_176840	100	440	0	0	914	475	0	698
ehcs0010P06.b	EHI_163530	100	195	0	0	224	418	1E-81	299
ehcs0010P24.b	EHI_105350	100	358	0	0	30	387	2E-162	569
ehcs0011E13.b	EHI_042930	100	169	0	0	169	1	1E-72	269
ehcs0011F01.b	EHI_197560	100	137	0	0	163	27	3E-53	204
ehcs0011H03.b	EHI_148780	100	143	0	0	1676	1818	2E-60	228
ehcs0011K14.b	EHI_104440	100	66	0	0	1147	1212	4E-24	106
ehcs0011M17.b	EHI_048730	100	70	0	0	16	85	5E-26	112
ehcs0011O04.b	EHI_001800	100	79	0	0	56	134	3E-30	126
ehcs0011A08.b	EHI_178560	100	643	0	0	647	5	0	1009
ehcs0011A12.b	EHI_054480	100	209	0	0	8	216	1E-91	332
ehcs0011A16.b	EHI_169840	100	391	0	0	17	407	3E-178	621
ehcs0011A22.b	EHI_105770	100	218	0	0	12	229	6E-96	347
ehcs0011B11.b	EHI_050960	100	416	0	0	21	436	8E-165	576
ehcs0011D07.b	EHI_087410	97,6	250	3	2	263	17	6E-85	310
ehcs0011D09.b	EHI_048670	100	228	0	0	711	484	2E-93	339
ehcs0011D11.b	EHI_111070	100	281	0	0	300	20	7E-126	446
ehcs0011D17.b	EHI_010510	99,69	646	2	0	784	139	0	917
ehcs0011E03.b	EHI_109690	99,51	615	3	0	865	251	0	971
ehcs0011E09.b	EHI_029030	100	204	0	0	230	27	3E-89	324
ehcs0011E10.b	EHI_182730	100	211	0	0	34	244	1E-92	336
ehcs0011E11.b	EHI_188850	100	637	0	0	9	645	0	898
ehcs0011E14.b	EHI_117850	100	276	0	0	436	161	2E-123	439
ehcs0011E23.b	EHI_136350	99,57	234	1	0	162	395	1E-102	369
ehcs0011F05.b	EHI_086230	100	266	0	0	29	294	2E-104	375
ehcs0011G15.b	EHI_092080	99,52	210	1	0	1367	1158	5E-61	232
ehcs0011G20.b	EHI_048420	99,73	374	1	0	385	12	8E-166	580
ehcs0011H07.b	EHI_042690	100	236	0	0	258	23	2E-104	375
ehcs0011I06.b	EHI_046260	98,16	326	0	1	1011	1336	5E-132	467
ehcs0011I10.b	EHI_188720	99,5	604	3	0	8	611	0	928
ehcs0011I19.b	EHI_066760	100	210	0	0	1	210	4E-92	334
ehcs0011K13.b	EHI_047800	100	579	0	0	628	50	0	919
ehcs0011L01.b	EHI_169620	97,66	128	3	0	131	4	4E-50	194
ehcs0011M02.b	EHI_010050	100	433	0	0	1518	1950	0	687
ehcs0011M23.b	EHI_000140	100	297	0	0	6	302	2E-133	472
ehcs0011O15.b	EHI_070680	99,86	697	1	0	1303	607	0	1093
ehcs0011O21.b	EHI_035670	100	198	0	0	392	195	2E-86	315
ehcs0012A01.b	EHI_008820	100	364	0	0	519	156	2E-161	565
ehcs0012A10.b	EHI_131130	99,84	626	1	0	1173	548	0	992
ehcs0012B10.b	EHI_184010	100	158	0	0	1226	1383	2E-67	252
ehcs0012C01.b	EHI_076270	100	157	0	0	701	545	5E-23	104

Gene ID et http://pathema.jcvi.org/ found by BLAST search.

**Table 2:** Bioinformatic search for hypothetical protein domains targeted by dsRNA interference.

CLONE	GENE ID (Pathema)	ORF (bp)	FRAGMENT (bp)	DOMAIN (amino acids) *	E-value
C3	EHI_053430	771	21–682	UBQ (185–255)	4.67e-01
D7	EHI_176840	1371	468–913	RNB (174–420)	4.70e-05
G1	EHI_044370	2046	1598–1892	Signal peptide (1–20)	
				SGA1 (301– 681)	4.00e-17
G5	EHI_148060	822	461–799	DUF850 (9–246)	9.40e-43
G7	EHI_178560	681	5–648	START (5–199)	6.40e-04

* Protein domains were identified by SMART search (http://smart.embl-heidelberg.de/).

UBQ: Ubiquitin homology; RNB: Catalytic domain of ribonuclease II;

SGA1: Glucoamylase and related glycosyl hydrolases;

DUF 850: This family consists of eukaryotic putative transmembrane proteins of unknown function; START: a lipid-binding domain

## Discussion

The data shown in the present report indicates that the use of bacterially-expressed dsRNA efficiently reduces gene expression in the protozoan parasite *E. histolytica*. This method is specific, affecting only the expression of the gene targeted whether the dsRNA was delivered via bacterial feeding or medium soaking. Our approach was mostly inspired by the RNAi approach established by the group of Dr. Andrew Fire in the nematode *Caenorhabditis elegans* [25] and also in RNAi studies with planarian fatworms [26]. Introduction of dsRNA into cells can be obtained by soaking these worms in solutions of dsRNA or feeding them with bacteria expressing dsRNA. The machinery allowing dsRNA degradation is subsequently triggered as a potent cell response where proteins binding dsRNA are central to induce the RNA interference (RNAi) phenomenon. During feeding, *E. histolytica* uses its capacities to phagocytise bacteria, although the mode of dsRNA delivery into the cytoplasm from the phagosomes is unknown. During soaking, dsRNAs enter into the amoeba by an unknown mechanism; potentially either by endocytic processes or by receptor- or transporter-mediated RNA entry. Identification of receptors or transporters for dsRNAs in *E. histolytica* would provide a new example of selective RNA uptake by cells, allowing direct and targeted gene expression modification by exogenous genetic material, as it was initially shown in *Caenorhabditis elegans* [27]. Whatever the dsRNA entry mechanism involved, we have shown that robust and reproducible RNAi phenotypes can be produced in *E. histolytica* upon contact with dsRNA synthesized in bacteria. The availability of *E. histolytica* genome allowed searching for orthologous genes containing the hallmark factors of the RNAi cascade. Surprisingly, no orthologous gene was found for the RNaseIII-family enzyme Dicer that catalyzes the cleavage of dsRNA segments into siRNA duplexes that are loaded onto the RNA-induced silencing complex (RISC) by the Argonaute component also responsible for cleaving target mRNA [28]. Nevertheless, an RNaseIII-like protein of *E. histolytica* was recently characterized and shown to use dsRNA as a substrate [20]. Moreover, other genes encoding orthologues of RdRP [29] and Argonaute [20] have also been reported to exist in *E. histolytica*.

Our group was the first to show that siRNA oligonucleotide duplexes induce specific gene silencing in *E. histolytica*, providing experimental evidence supporting the existence of an RNAi pathway in this parasite [21]. This is also consistent with other gene silencing studies for *E. histolytica* using episomally-expressed anti-sense and shRNA [22], [23]. Nevertheless, technical disadvantages related to the former methods encouraged us to standardize a method that could be used systematically for a large number of targets. The feeding method with dsRNA expressing bacteria used for this work, took advantage of the phagocytic capabilities of *E. histolytica*. Interestingly, this method was shown to be a direct and cost-effective way to downregulate the housekeeping gene β-tubulin (Fig. 2). These initial observations using bacterial feeding encouraged us to target other *E. histolytica* genes.

The *E. histolytica* virulence factor KERP1 [12], [30] that was previously characterized as a tightly regulated gene, was an interesting and challenging target to further assess the effectiveness of gene silencing mediated by bacterial dsRNA. A moderate but significant reduction of KERP1 protein abundance was found using bacterial feeding, however, no significant *kerp1* transcript reduction was observed. This observation correlates with our previous results, showing that *kerp1* anti-sense RNA transcription in *E. histolytica* does not lead to a reduction of the full-length, endogenous mRNA abundance, unless amoebae are stressed [12].

The inhibitory effect of dsRNA was also verified in another series of experiments using soaking as a delivery method. A more consistent transcript and protein downregulation was found with soaking even though the amount of purified dsRNA that was used was similar to unpurified bacterial dosage used in the feeding experiments. Differences in the entry of dsRNA might account for the better efficacy of dsRNA soaking. These results are consistent with other RNAi studies comparing the use of feeding and soaking as delivery methods in parasites [31]. Another advantage using the medium soaking was that no changes in the growth behaviour of the trophozoites were observed using unrelated dsRNA, probably due to conditions bypassing the presence of unspecific bacterial material and bacteriostatic drugs used for the feeding. The experimental conditions established so far for the inhibitory effects of dsRNA allowed us to confirm for the first time *in vitro* the previously proposed role of the virulence factor KERP1 [30]. The inhibition of KERP1 by soaking showed its involvement in parasite adhesion to human cells and concomitant cytopathogenic capabilities. Moreover, RNAi conditions were also useful for the screening of growth-related factors. Potentially interesting genes were for the first time described, opening alternatives for studies on the phenomenon of lipid–cell transfer and RNA degradation of ubiquitination in this parasite.

At this point, the molecular mechanism providing effectiveness of RNAi in *E. histolytica* is not known; however, the methods we describe here are feasible, affordable and therefore should provide greater general utility than previously published methods. Both methods we described are efficient and each of them has its own advantages. The soaking method is highly reproducible, does not require antibiotics, phagocytosis or the presence of unspecific bacterial material. The feeding method is fast, inexpensive and easy to perform; we already built a library available for the scientific community, allowing immediate and cost-effective experiments and screenings. These methods will improve gene knockdown studies that, in combination with recent developments in instrumentation and image analysis, should enable the use of high-throughput screening approaches to elucidate the pathogenic capabilities of *E. histolytica*.

## Materials and Methods

### Bacterial Strain, Cells and Culture Conditions

RNAseIII-deficient *Escherichia coli* strain HT115 (rnc14:: ΔTn10) [32] was grown in LB broth for genetic engineering procedures or 2YT broth for the expression of dsRNA, both containing antibiotics ampicillin (100 µg/ml) and tetracycline (10 µg/ml).

The pathogenic *E. histolytica* strain HM1:IMSS was cultured axenically in TYI-S-33 media at 37°C, as previously described [33].

For the bacterial feeding experiments, *E. histolytica* trophozoites were cultured in F-TYI-S-33 medium (TYI-S-33 supplemented with 34 µg/ml chloramphenicol, 100 µg/ml ampicillin and 10 µg/ml tetracycline).

For growth kinetics in culture tubes, adherent trophozoites were detached at indicated time with ice-cold incubation for 5 min and cells were counted using a Malassez slide and trypan blue exclusion.

Human liver sinusoid endothelial cells (LSEC) were cultured in RPMI-glutamax medium (Gibco BRL) supplemented with 10% heat-inactivated fetal calf serum, 100 U/ml penicillin and 100 g/ml streptomycin, at 37.5°C in a 5% CO_2_ incubator, as previously described [34].

### Plasmid Constructs for the Expression of dsRNA

For the construction of dsRNA-expression vectors, DNA fragments at the 5′ end of the *E. histolytica* genes (753 bp for beta-tubulin, GenBank accession n°: EHI_049920; 388 bp for KERP1, GenBank accession n°: EHI_098210) and green fluorescent protein (GFP) full coding sequence (GenBank accession n°: U73901) were amplified by PCR using specific primers (see Table S1) and subcloned into the TA-cloning vector pCR2.1-TOPO (Invitrogen). DNA inserts were excised from TA-cloning constructs with restriction enzymes (*Kpn*I and *Bam*HI for beta-tubulin and GFP and; *Eco*RI for KERP1) and cloned into the MCS of L4440 plasmid vector [25] that is bidirectionally flanked by T7 promoters. The resulting plasmid constructs L4440-beta-tubulin, L4440-KERP1 and L4440-GFP were verified by restriction analysis and DNA sequencing.

### Bacterial Transformation and Expression of dsRNA

HT115 competent cells for bacterial transformation were prepared by the rubidium chloride method [35]. Competent cells were transformed with recombinant plasmid DNA by heat shock pulse at 42°C for 60 sec. After recovery on LB media at 37°C for 1 hr, cells were spread on LB-agar plates containing ampicillin-tetracycline. Isolated bacterial colonies were obtained after overnight culture at 37°C.

Bacterial expression of individual dsRNA species was carried out in standard culture flasks filled with 1/10 of total capacity, incubated at 37°C with shaking at 220 rpm or in static 96 deep well plates (Axygen) filled with 1.5 ml of culture broth and sealed with breathable sealing film. Overnight bacterial cultures of isolated colonies were diluted 1/50 in 2YT broth supplemented with ampicillin/tetracycline and cultured at 37°C to an optical density of 0.6 at 600 nm, (∼5 hr with shaking or 36 hr for static deep well plate culture). Supplementary ampicillin/tetracycline was added as above and bidirectional transcription of complementary RNA strands was induced with 2 mM IPTG during 4 hr at 37°C with shaking or overnight for deep well plates.

### Trophozoite Feeding with dsRNA-Expressing Bacteria

Glass culture tubes or plastic flasks containing F-TYI-S-33 were inoculated with trophozoites freshly collected from ∼90% confluent culture to obtain a final density of 2 to 3.5×10^4^ cells (as indicated for each experiment) per 14-ml tube for growth kinetic assays or a ∼10% confluence (10^5^ amoebae) for other experiments, and incubated at least 1 hr at 37°C before bacterial feeding. For each condition, a bacterial inoculum prepared in F-TYI-S-33 was added to the trophozoite culture to obtain a final ratio of 10^4^ bacteria per amoeba. Cellular density of bacterial inoculums was determined assuming that an optical density of 1 at 600 nm corresponds to 10^8^ bacteria/ml. Trophozoites fed only once with dsRNA-expressing bacteria were incubated at 37°C during indicated time of culture.

### Statistical Analysis

Mean values from experimental replicates were analyzed by using a paired *t*-test to determine between-groups significance. * *p*-values <0.05 were considered significant; ** stands for *p*-values <0.01, *** for *p*-values <0.001.

### Purification of Bacterial dsRNA

Fractions of bacterially-expressed dsRNA species were prepared using a protocol that was adapted for large-scale purification (12–20 µg/ml of induction) or 96 well microtiter plate purification (V-shape wells, Nunc) [25]. For each bacterial clone, cells were induced for the expression of dsRNA and bacterial pellet was resuspended in 1 M ammonium acetate, 10 mM EDTA to 1/20 of initial induction volume. After adding the same volume of phenol∶chloroform ∶isoamyl alcohol (25∶24∶1) pH 8.0, samples were mixed, incubation at 65°C for 15 min and centrifuged at 10,000 *g* for 15 min (for plates: 3,200 *g* for 30 min). The upper phase was transferred to fresh tubes or wells and mixed with equal volume of isopropanol, incubated 30 min at −20°C, then centrifuged at 12,000 *g* for 30 min (for plates: 3,200 *g* for 2 hr). Nucleic acid pellet was washed with 70% ethanol, air-dried, then resuspended in 1/100 of initial induction volume with boiling nuclease-free water, then supplemented with Turbo DNase buffer (Ambion) and incubated 30 min at room temperature. Nucleic acid solution was treated with 0.4 U/µl of Turbo DNase and 0.2 µg/µl of RNase A (Ambion) at 37°C for 1 hr. After adding 1 volume of phenol:chloroform:isoamyl alcohol, samples were mixed and centrifuged at 12,000 *g* for 15 min (for plates: 3,200 *g* for 30 min). Upper phase was collected, mixed with 0.5 volume of 7.5 M ammonium acetate and 1 volume of isopropanol, and then centrifuged at 12,000 *g* for 30 min. Each pellet was washed twice with 70% ethanol, air-dried and resuspended in 1X TE. Samples were incubated for 1 min at 90°C and cooled down at room temperature for 30 min. Double-stranded RNA fractions were analyzed by agarose gel electrophoresis and concentration determined by spectrophotometry.

### Trophozoite Soaking with Bacterial dsRNA

Trophozoites that were seeded in culture flasks or tubes using an initial density identical to the feeding experiments were treated with purified fractions of bacterial dsRNA species that were directly added into culture media to a final concentration of 5, 25 and 50 µg/ml. Soaked trophozoites were incubated at 37°C during indicated time of culture.

### Quantitative Real-Time PCR

Trophozoite RNA was purified by using TRIzol method (Invitrogen) as previously described [12]. After primer-specific reverse transcription, qRT-PCR was performed (primers presented in Table S1) and analyzed according to a published method [12]. Three independent biological samples were analyzed (*n* = 3); for each of them, data from two 10-fold dilutions of DNA matrix, each repeated in triplicates, were averaged.

### Antibodies

A rabbit anti-beta-tubulin polyclonal antibody was obtained from Eurogentec (Belgium) by immunization with two synthetic peptides with sequences derived from the amino acid sequence of *E. histolytica* beta-tubulin (amino acids 50 to 64: FSESSTKRYVPRSIN; and amino acids 324 to 338: DVEEQLYKIREKNPD). Immunoglobulin fraction from polyclonal serum was purified using protein G affinity chromatography (GE Healthcare Bio-Sciences) and the specificity was verified by western blot analysis using recombinant fractions of *E. histolytica* alpha, beta and gamma tubulin (not shown). An anti-KERP1 monoclonal antibody used for this work was obtained as described elsewhere [12].

For western blotting analysis, the amount of proteins was normalized using an anti-actin C4 monoclonal antibody and secondary HRP-antibodies (MP Biomedicals) as previously described [12].

### Western Blot Analysis

Trophozoites from feeding or soaking experiments were collected to prepare crude extracts using a protease inhibitor cocktail (Roche) as previously described [12]. Crude extracts (10^4^ cells/lane) and a calibration curve of different protein amounts of the reference sample were resolved by SDS-PAGE. Electrophoresed proteins were transferred to nitrocellulose membranes and probed with specific antibodies and ECL Plus reagent (GE Healthcare Bio-sciences) for chemiluminescence detection [12]. Semi-quantitative analysis of light emission from probed nitrocellulose membranes was carried out using Gel doc apparatus and Quantity one software (BioRad).

### Cell Adhesion Assays

Adhesion of trophozoites to human liver sinusoid endothelial cells (LSEC) was determined using fluorescent micrographs and image analysis from in vitro assays performed in flat-bottom 24-well microtiter plates (TTP) [36]. Briefly, trophozoites previously treated with purified dsRNA and untreated trophozoites, were stained with 2.5 µM Cell Tracker Orange CMTMR (Invitrogen) at 37°C for 30 min and inoculated in triplicate to a final density of 10^5^ parasites/well together with formaldehyde-fixed LSEC monolayers, as previously described [36]. After 30 min at 37°C in 5% CO_2_, wells were washed with warm phosphate buffer saline (PBS) at 37°C and fixed with 5% formaldehyde for 30 min, then washed again. Micrographs from microtiter plate wells (9 fields per well) were obtained using confocal apotome microscopy (Zeiss) and trophozoites were manually counted using ImageJ software's plug-in “cell counter” (http://rsb.info.nih.gov/ij). The experiment was repeated thrice; the results from the triplicates on each plate were pooled and expressed as relative adhesion index using the following formula [(mean number of adherent trophozoites) / (mean number of adherent trophozoites in the untreated conditions from the 3 experiment repetitions)].

### Cytophatogenicity Assays

Trophozoite cell cytopathogenic activity was tested against LSECs as target cells, using a fluorescence assay that was modified from previously described method [36]. The assay was performed in flat-bottom 24-well microtiter plates (TTP). Trophozoites previously treated with purified dsRNA and untreated trophozoites, were seeded in triplicate to a final density of 10^5^ parasites/well together with BCECF-labeled LSEC monolayers [37]. After 1 hr at 37°C in 5% CO_2_, culture media supernatant was removed, wells washed twice with warm PBS at 37°C and fresh media was added. The fluorescence was read for all of the wells directly by the Fluoroskan Ascent (Thermo Scientific) at excitation/emission wavelengths of 485/538 nm. Fluorescence data was used to calculate raw cytopathogenicity rate according to the formula [(background fluorescence – test fluorescence) / (background fluorescence – maximum cytopathogenicity)]. Background fluorescence represents fluorescence from LSECs in medium alone, and maximum cytopathogenicity is the fluorescence from LSECs totally lysed in medium plus 1% Triton X-100, each measured in three replicate wells. The experiment was repeated thrice; the results of the triplicates were averaged and expressed as the index of cytopathogenicity, from the formula [(raw cytopathogenicity rate) / (raw cytopathogenicity rate in untreated conditions from the 3 experiment repetitions)].

### Repertoire of dsRNA Expressing Clones

Synthesis of double-stranded cDNA from *E. histolytica* poly(A)-mRNA was carried out using oligo-(dT) primer, Superscript II Retro-Transcriptase and DNA polymerase I (Ambion). Fragments of cDNA higher than 300 bp were purified by size-exclusion chromatography column, phosphorylated and ligated to EcoRV-digested and dephosphorylated L4440 plasmid vector. HT115 competent cells were transformed with ligation reaction as described above. Individual colonies from bacterial transformation were transferred from LB-agar plates to microtiter plate wells containing LB broth - ampicillin/tetracycline using an automated system (TECAN Genesis workstation), and grown overnight at 37°C for in plate plasmid DNA purification and sequencing analysis. Full sequence of plasmid inserts from one thousand bacterial clones was analyzed in silico (using BLAST programme) and a list of 408 bacterial clones carrying amoebic coding sequences was obtained. All cDNA sequencing data and blast results were deposited at GeneBank (http://www.ncbi.nlm.nih.gov/Genbank/index.html) with the accession number GT128032 to GT128439. Among positive clones, a repertoire of 85 clones carrying distinct coding sequences for hypothetical proteins of unknown function was selected for the 96-well microtiter plate purification of dsRNA and growth phenotype screening (Table 1).

### Microtiter Plate Screen

A high-throughput screening for trophozoite growth phenotype after treatment with distinct species of dsRNA directly soaked in culture media supernatant was carried out in flat-bottom 96-well microtiter plates (Greiner Bio-One). Trophozoites freshly collected were seeded in plate wells to a final density of 600 parasites/well [38]. After 1 hr at 37°C in a Genbag anaer (BioMérieux), plate wells were individually soaked with a repertoire of 85 distinct dsRNA species that were previously purified as above, using ∼1 µg of dsRNA per well in 20 µl. After 24 and 48 hr of culture, light micrographs from microtiter plate wells were obtained for the identification of wells showing a reduction of trophozoite numbers, using as a reference untreated or GFP-dsRNA treated trophozoites.

## Supporting Information

Table S1 Oligonucleotide primers for cloning and qRT-PCR.
(0.03 MB XLS)
